# Epstein Barr virus DNA analysis in blood predicts disease progression in a rare case of plasmablastic lymphoma with effusion

**DOI:** 10.1186/1750-9378-8-28

**Published:** 2013-07-23

**Authors:** Anna Friis, Börje Åkerlund, Birger Christensson, Katarina Gyllensten, Anna Aleman, Jie-Zhi Zou, Ingemar Ernberg

**Affiliations:** 1 Department of Microbiology, Tumor and Cell Biology, Karolinska Institutet, SE-171 77 Stockholm, Sweden; 2 Unit of Infectious Diseases, Department of Medicine, Karolinska University Hospital, Huddinge, SE-141 86 Stockholm, Sweden; 3 Department of Laboratory Medicine, Karolinska University Hospital, Huddinge, SE-141 86 Stockholm, Sweden; 4 Venhälsan, Södersjukhuset, SE-118 83 Stockholm, Sweden

**Keywords:** EBV, HIV-1, HHV-8, DNA load, Plasmablastic lymphoma

## Abstract

**Background:**

In HIV-1-infected patients a long lasting CD4+ cell decline influences the host-EBV balance and thereby increases the risk for EBV related malignancies. In spite of a world-wide access to combination antiretroviral therapy (cART) there are still a considerable number of HIV-1-infected patients who will develop severe immunodeficiency. These undiagnosed HIV-1 infected patients, so called late testers, demonstrate an increased lymphoma risk, compared to patients diagnosed early. Consecutive individual screening for EBV DNA-load in late testers might be a useful predictor of emerging EBV-malignancy.

**Methods:**

Patient biopsies and ascites were analyzed morphologically, by immuncyto-histochemistry and in-situ hybridization. Viral DNA and RNA load were quantified by PCR. Cell lines from primary tumor and from ascites, were established *in vitro* and further analyzed.

**Result:**

We here report on a case of EBV-positive lymphoma in an AIDS patient, first presenting with pleural effusion and ascites and was thus initially considered a primary effusion lymphoma (PEL) but was later diagnosed as a plasmablastic lymphoma (PBL). The patient had responded to cART with undetectable HIV-RNA and increased CD4 cell count one year prior to lymphoma presentation. At the time of lymphoma diagnosis the HIV-RNA values were <50 RNA-copies per mL blood (undetectable) and the CD4-positive cell count 170 ×10^6^/L. The lymphoma was CD45-negative and weakly CD22- and CD30-positive. The patient had a history of Kaposi sarcoma and HHV-8 seropositivity. The lymphoma biopsies, and three cell lines derived on different occasions from the tumor cell effusion, were all EBV-positive but HHV-8 negative.

A noticeable EBV-DNA load decline was observed during the remission of the lymphoma following CHOP-therapy. The EBV-DNA load increased dramatically at the time of recurrence.

**Conclusion:**

EBV DNA load might be useful in monitoring the effect of lymphoma treatment as well as in estimating the risk of EBV-associated lymphoma in HIV-1 infected patients with pronounced immunosuppression.

## Background

EBV infects the vast majority of humans and resides latently in B-cells. The virus carries genes that can induce and maintain mature B cell growth. In immunocompromised post-transplant and HIV-infected patients the virus may cause lymphoproliferative or malignant diseases. Although EBV negative lymphoid malignancies have been described in HIV-infected persons EBV has a strong association with a wide range of B-cell lymphomas in HIV-carriers including Burkitt’s lymphoma (BL), Hodgkin lymphoma and Diffuse Large B-cell lymphomas [1].

Plasmablastic lymphoma (PBL) is a rare aggressive subtype of non-Hodgkin lymphoma (NHL) most commonly localized to mucosal tissues such as the oral cavity, but may also be manifest in other organs such as liver, breast or bone [2]. Morphologically and immunophenotypically PBL have some similarities to primary effusion lymphomas (PEL) and both lymphomas are EBV-positive and associated with immunodeficiency, most frequently due to HIV-1 infection. Body cavity effusion to pleura or abdomen, that is the hallmark of PEL, is much less frequently seen in PBL. Moreover, PEL in virtually all cases is HHV-8-positive. In contrast PBL is rarely HHV-8 positive [3].

Furthermore, PBL is CD45-negative, while PEL is CD45-positive. Both PBL and PEL display markers associated with plasma cell differentiation such as CD38 and CD138 and usually express cytoplasmic immunoglobulin and may be variably positive for CD30. Although most cases of PBL occur in AIDS-patients with profound immunodeficiency, HIV-1 negative cases have also been reported [3-8].

In the era of modern combination antiretroviral therapy (cART) there has been an impressive reduction of AIDS-related opportunistic infections and lymphomas. However, HIV-1 patients still suffer an increased risk for NHL [9] and the risk for development of NHL might be considerable in patients with a late diagnosis of HIV- infection and with severe immune dysfunction – so-called late testers [10,11]. As HIV-1 infection induces a new viral set point between host and EBV [12] several immune modulating factors e.g. CMV, bacterial translocation [13] or even vaccination [14] might trigger lymphoma genesis.

## Results

### Clinical report

When the patient was diagnosed with HIV-1 the CD4+ cell count was 170 × 10^6^/L. In a malignancy assessment the same year endoscopic biopsies were taken from the gut mucosa. By routine histopathological analysis the biopsies showed signs of chronic inflammation and macrophage infiltration, but no lymphoma. Bone marrow aspiration was also performed with May-Grunewald stained smears showing immature cells of the myeloid series and many lymphoid cells (44%), including cells of lymphoplasmocytoid/plasmacell type. Staining for kappa and lambda light chains gave no evidence for clonal B-cell proliferation. The findings were suggested to represent a polyclonal B-cell hyperplasia without suspicion of lymphoma.

**Figure 1:**
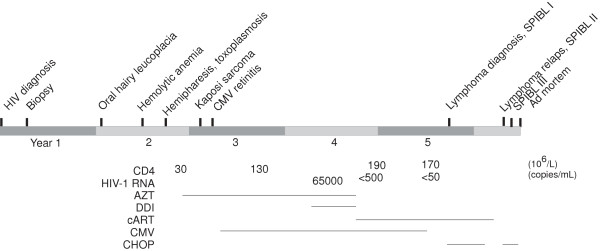
**Schematic presentation of patient HIV-1 disease related manifestations. **Important clinical manifestations and time point of histopathologic analysis of Figures 2, 3, 4 and 5 are shown. SPIBL I, II, III: tumor tissue and ascites derived cell lines were established on three occasions.

Oral hairy leukoplakia was diagnosed early the year after HIV-diagnosis and the same year the patient developed hemolytic anaemia (Figure 1). The anaemia resolved with steroid treatment and the patient was subsequently maintained on a low dose of prednisolone. By the end of the second year after diagnosis of HIV infection he contracted hemiparesis and a CT scan showed a contrast enhancing lesion in the parietal left brain hemisphere, suggestive of toxoplasmosis infection. The patient improved on treatment with anti toxoplasmosis drugs and continued this suppressive therapy.

In December the second year antiretroviral treatment with azidothymidine (AZT) was initiated, and shortly thereafter a conjunctival Kaposi lesion was removed. In February the following year (year 3; Figure 1) the patient had a CD4+ cell count of 30 × 10^6^/L, and CMV-retinitis was diagnosed and treated with altering foscarnet and ganciclovir. During the fourth year HIV-RNA values increased to 65 000/mL prompting a change in the anti-retroviral treatment to AZT and didanosine. Second half of fourth year cART was introduced (AZT, lamivudine, and indinavir). After cART introduction, including a protease inhibitor, HIV-RNA was <500/mL, and remained undetectable during the following two years. The patients CD4 cell count went from 30 × 10^6^/L in second half of year two after HIV-1 diagnosis to 190 × 10^6^/L after introduction of cART year four. The suppressive CMV-treatment was withdrawn the same year (year 4).

Soon after withdrawal of CMV treatment the patient developed night sweats and elevated lactatdehydrogenase (LD), and one month later ascites. CT scan revealed a mass in the gastric wall and pleural effusion. An initial cytological analysis of the effusion suggested a primary effusion lymphoma (PEL). A biopsy from the tumor in the gastric wall confirmed the presence of an EBV-positive Non-Hodgkin lymphoma with immunoblastic features considered to be a manifestation of PEL.

After diagnosis of the lymphoma cytostatic treatment with cyclophosphamide, hydroxydaunorubicin (Doxorubicin), vincristine (Oncovin), prednisolone (CHOP), and intrathecal methotrexate was initiated and the ascites disappeared within four days. At the time of lymphoma treatment Rituximab was not yet approved and was therefore not a treatment option. The patient continued with cART and suppressive toxoplasmosis treatment. The CHOP treatment was repeated every 3rd week and the patient completed six treatment cycles in total. Before every CHOP treatment blood was drawn for EBV-DNA analysis. EBV-load was also measured on three occasions the month after completing CHOP treatment. The remission of the tumor mass of the gastric wall was confirmed with a CT-scan and the patient did clinically well. Then, one month after the final CHOP course, ascites reappeared together with pleural effusion. Reintroduced cytostatic treatment now only had marginal effect on the lymphoma. Pleural effusions were evacuated twice. The patient died four and a half years after HIV-1 diagnosis due to progression of the lymphoma.

At autopsy lymphoma manifestations were observed in spleen, liver, lymphoid organs and lymphomatous dissemination to pleura and peritoneal cavity. A small cerebral mass in globus pallidus was also found, but this was not further analyzed to rule out lymphomatous spread to the central nervous system lymphoma or a lesion related to the patient’s earlier diagnosed cerebral toxoplasmosis.

### Morphologic, immunohistochemical and flow cytometric analysis

Retrospective in situ hybridization of the patient’s early Kaposi manifestation did show EBER-expression in some (probably lymphatic) cells.

**Figure 2:**
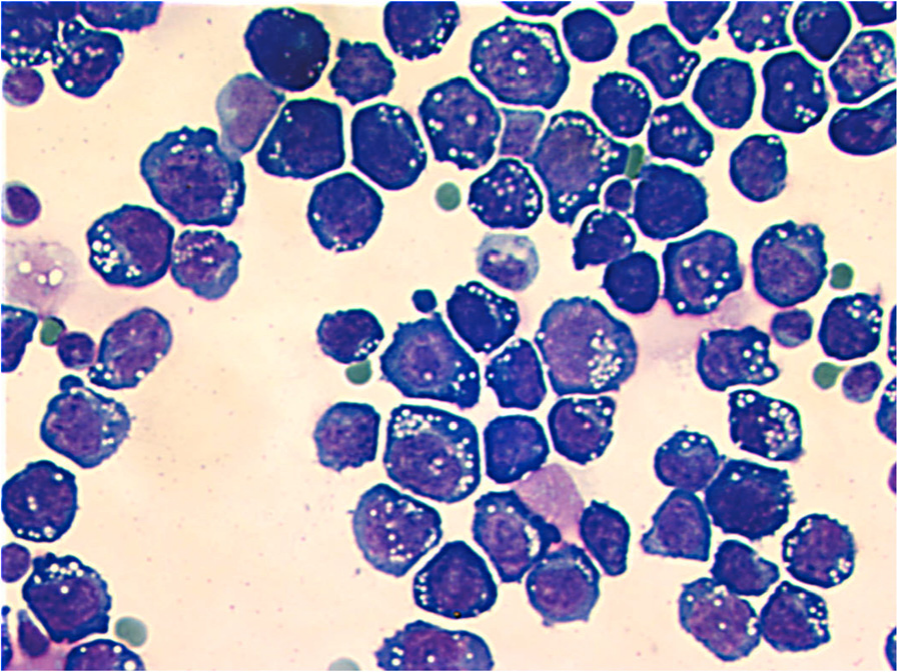
**Cytological examination of cells from abdominal ascitic fluid. **Stained with Giemsa.

**Figure 3:**
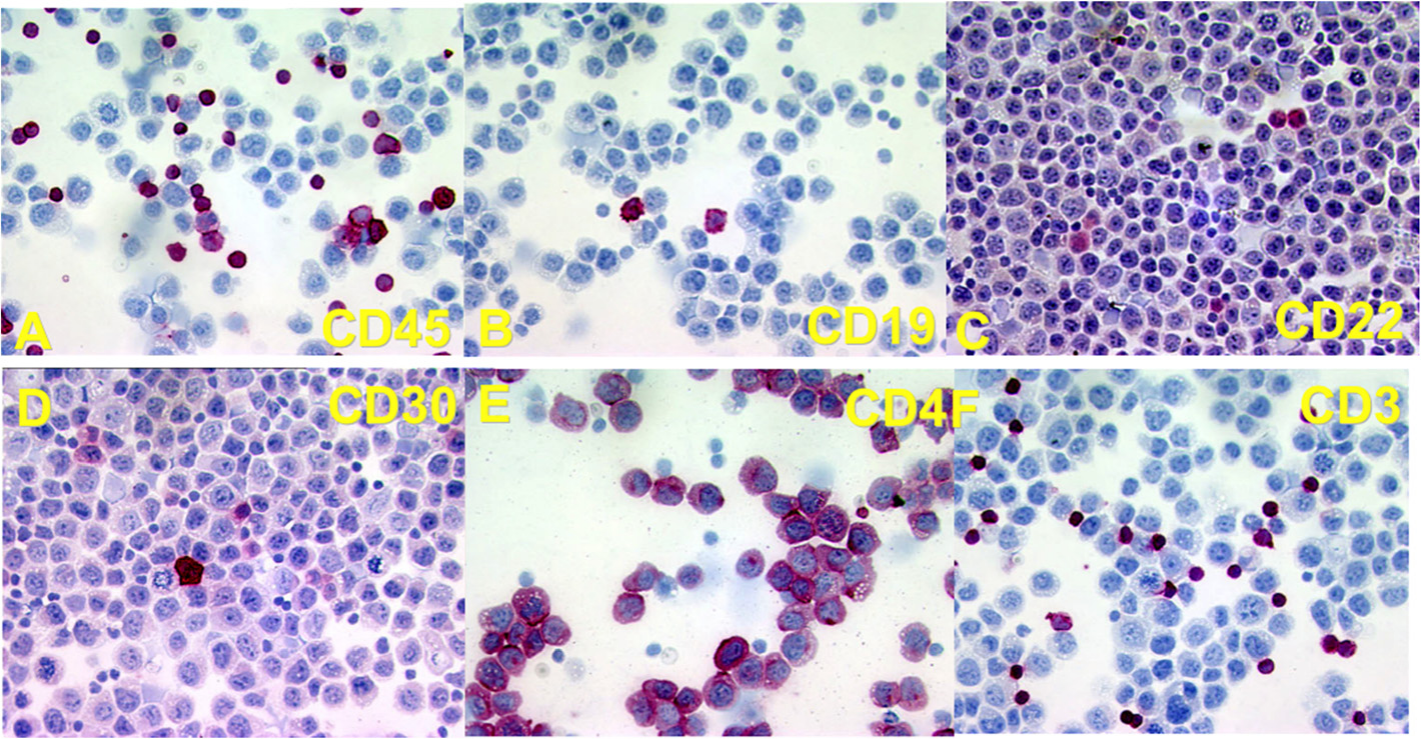
**Immunohistochemistry of cytospin from abdominal ascites. **Staining for CD45 **(A)**, for CD19 **(B)**, CD22 **(C) **CD30 **(D)**, CD4 **(E) **and CD3 **(F)**.

When the patient presented with a gastric tumor and ascitic and pleural effusions, cytological examination of the ascitic fluid showed a population of dispersed large blastic cells with a high frequency of mitoses and partly vacuolated dark blue cytoplasm. The tumor cells were morphologically highly reminiscent of blastic lymphoid cells, with features of immunoblastic plasmablastic differentiation (Figure 2). By immunochemical staining of cytospins from the ascitic fluid of tumor cells were negative for CD45, as well as for CD19 and CD79A. The tumor cells were only weakly positive for CD22 and CD30, but strongly positive for CD4 (Figure 3). By immunocytochemistry strong staining for EBV-LMP1 was also seen in some of the blastic tumor cells. Small lymphocytes positive for CD3, CD5 and CD8 were seen at low frequency both by flowcytometry (around 10%) and by immunocytochemistry. A cell line derived from the effusion showed a lymphoblastoid appearance.

**Figure 4:**
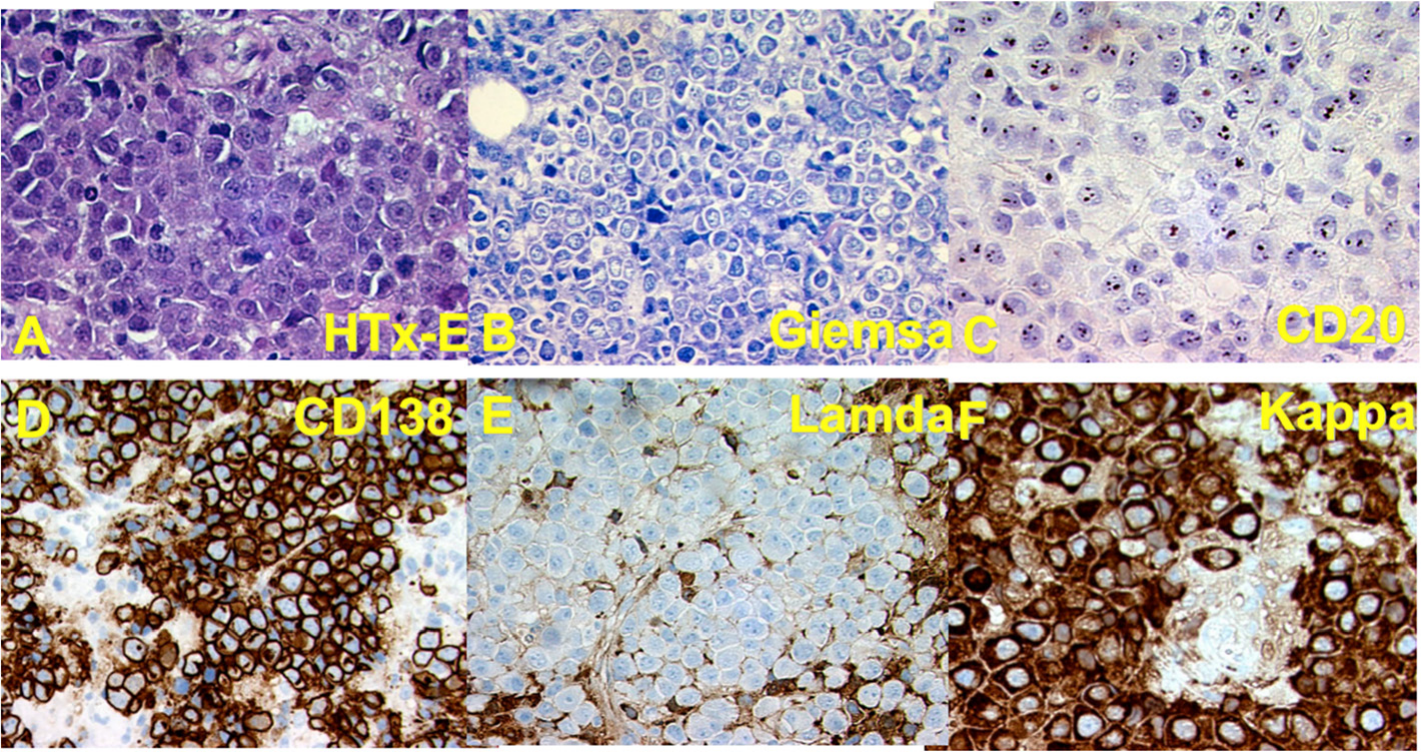
**Immunohistochemistry staining of tissue sections of a core biopsy of the abdominal tumor from ventricular wall. **Hematoxylin- eosin **(A) **and Giemsa staining **(B)**, CD20 **(C)**, CD138 **(D)**, lambda **(E) **and kappa **(F)**.

A trans-abdominal core biopsy was subsequently obtained from the extra nodal tumor in the gastric wall, which was prepared for flow cytometry and histopathology and molecular analysis. The routine hematoxylin-eosin (H-E) staining showed an infiltration of a large cell malignant lymphoma with immunoblastic/plasmablastic features, but without any mucosal component or lymph node structures in the specimen (Figure 4). The morphology was considered compatible with the findings in cytological specimens from the ascitic fluid and thus initially suggested to be a solid tumor component of a PEL. However, later investigations showed negative results for HHV8 LANA1 antigen staining. Additional immunhistochemical stainings revealed negativity for CD45 and CD20, but a clonal cytoplasmic Kappa staining (Figure 4). Thus, the lymphoma was reconsidered to better fulfill the criteria for a PBL with pleural effusion and ascites already at diagnosis than a PEL. Further immunohistochemistry revealed a weak positive staining for CD74, the HLA-DR associated invariant chain, negative staining for cytokeratins, the melanoma antigen HMB45 and CD34. Tumor cell staining for EBNA 2 was negative, whereas LMP1 was strongly positive in a few cells. Approximately 90% of the tumor cells were positive for Ki67 (data not shown).

**Figure 5:**
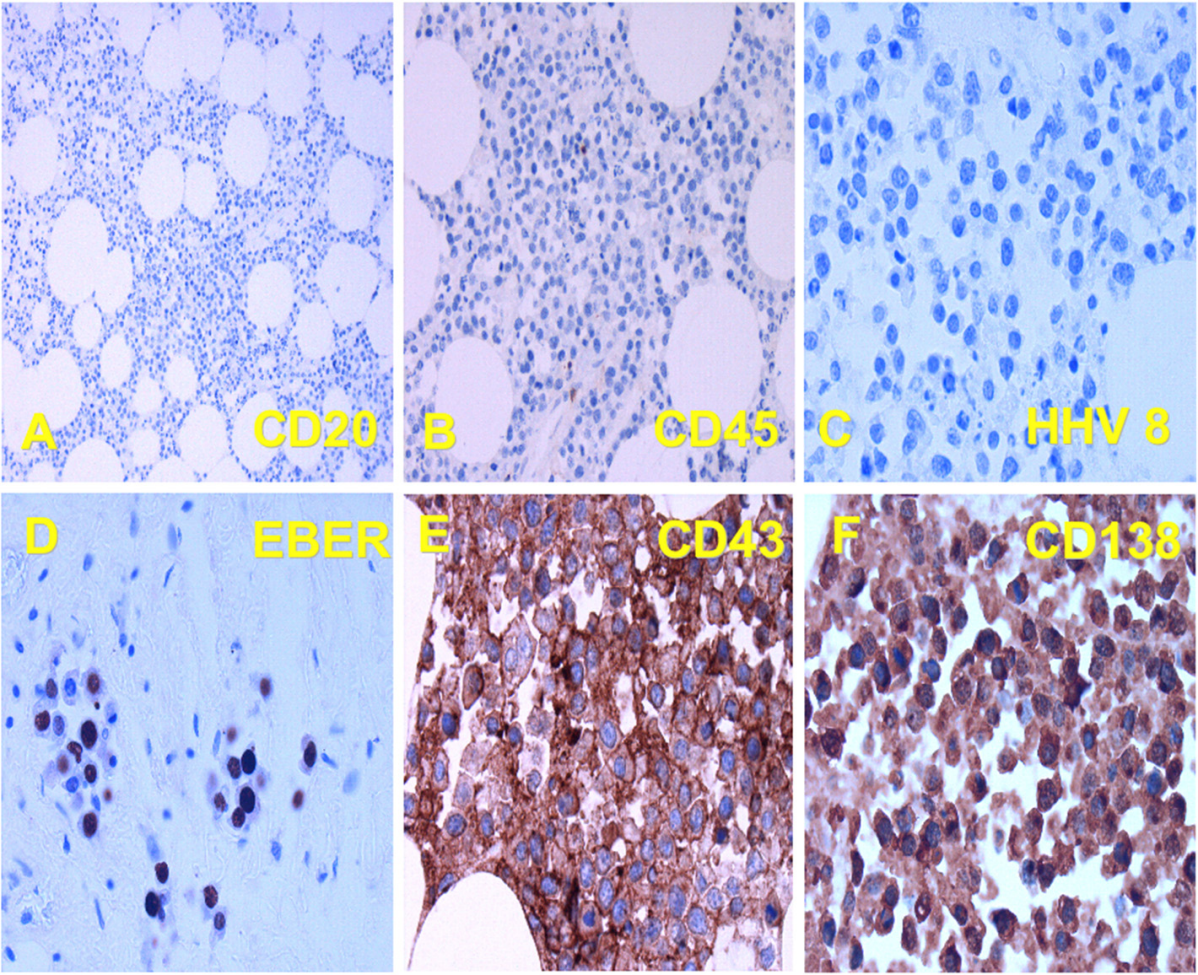
**Immunohistochemistry of mediastinal tumor material post mortem. **Stained with antibodies to CD20 **(A)**, CD45 **(B)**, HHV-8 **(C)**, and in situ probing for EBV EBER **(D)**, CD43 **(E)**, leukocyte membrane antigen and CD138 (syndecan 1; **F**).

Additional analysis was performed on autopsy material. Immunohistochemical analysis on sections was positive for CD43 and CD138 (syndecan 1; Figure 5) CD38 and Mum1. In-situ hybridization for nuclear EBV RNA (EBER) was positive, while HHV-8, CD20, PAX5 and CD45 all were negative by immunohistochemistry (data not shown).

This case illustrates a diagnostic dilemma. The initial finding was that of a blastic EBV-positive lymphoma primarily detected in effusions, lacking obvious B- and T-cell markers except CD4 but showing evidence of plasma cell differentiation as indicated by positivity for CD38 and CD138. Based on this information a diagnosis of PEL was originally suggested. However after further investigations showing clonal kappa positivity but HHV8 and CD45 negativity the case was finally considered to better fulfilling the criteria for a PBL.

Although effusions to the pleura and abdomen are rare features of PBL, they are the hallmarks of PEL. Conversely extra-nodal solid organ involvement, a feature of PBL is rarely seen in PEL, thus emphasizing the diagnostic dilemma.

The fact that the patient's lymphoma was CD45-negative, HHV-8-negative and EBV-positive became decisive for the final classification.

### EBV- DNA analysis

**Figure 6:**
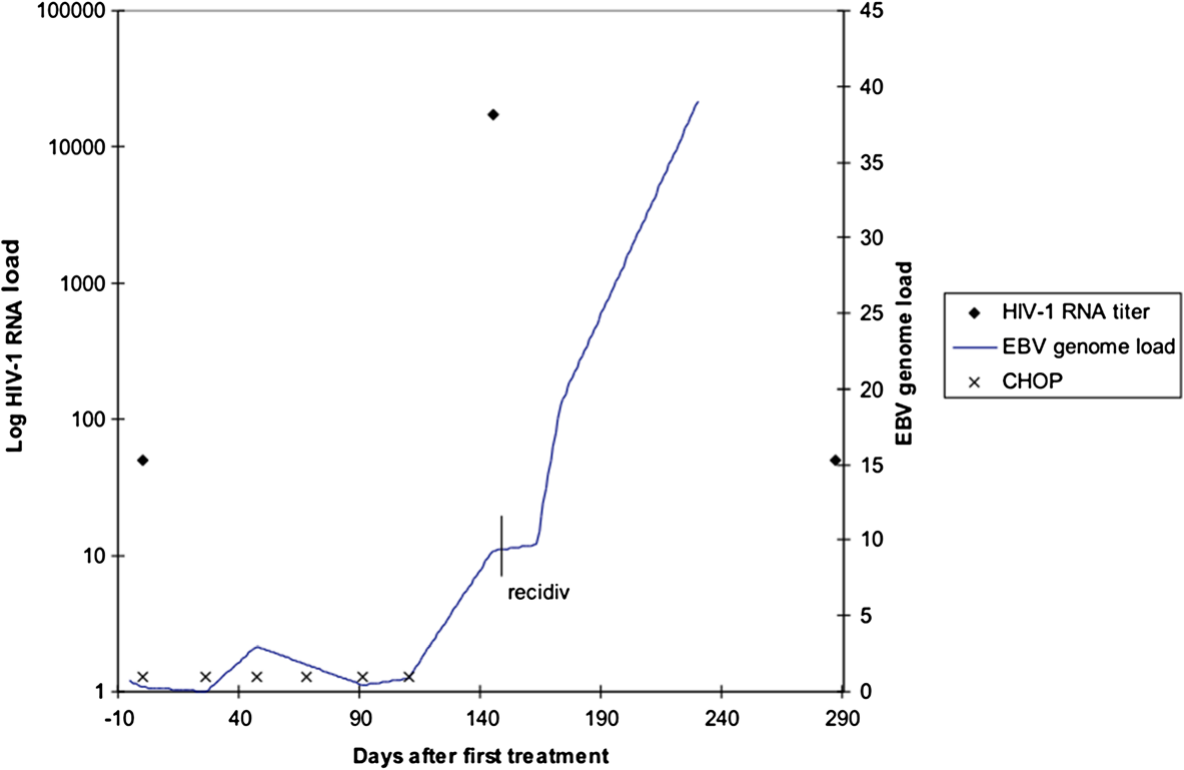
**EBV genome load, HIV-1 RNA titer, treatment events and relapse. **Time line illustration of measured parameters, treatment events and clinical findings. Time 0 is equal to first blood sampling for HIV-1 RNA analysis and EBV genome load measurement. HIV-1 RNA titers are illustrated with rhombic symbol. EBV genome load development is illustrated by solid line, and CHOP treatment events indicated by x's.

A noticeable decline of EBV-DNA load was observed during remission of the lymphoma following CHOP-therapy. The EBV-DNA load then increased dramatically at the time of recurrence (Figure 6, Table 1).

**Table 1:** Quantitative EBV-PCR in B-cells

**Time point (week)**	**EBV-DNA-PCR (genomes/1000 B-cells)**	**Treatment**	**HIV-RNA (copies per mL blood)**	**Comments**
0	0.75			
1	0.29	CHOP	<50	remission
5	0.0084	CHOP	50	
6	3	CHOP		
9		CHOP		
13	0.43	CHOP		
16	0.89	CHOP		
17	9.3		17,000	relapse
23	9.7			
24	19	Foscavir		
26		Tuxol		
32	39			
41			<50	

### Cell lines

Cell-lines were successfully derived from the patient lymphomas on three different occasions. One was established from ascites infusion during the first manifestation before therapy (SPIBL-I; second half of year 4; Figure 1) and two from pleural effusions after the relapse (SPIBL-II & III; late first half of year 5; Figure 1). These cell lines were characterized for EB-viral gene-expression and presence of EBV-, HHV-8- and HIV-1-genomes, morphology, karyotype and immunoglobulin-gene rearrangement (Tables 1 &2). They were all of B-lymphocyte origin expressing cell surface immunoglobulins. They carried EBV-DNA, but no HIV- or HHV-8-genomes were detected. Most conspicuously their phenotypes were distinctly different. SPIBL-I showed morphologic features of a lymphoblastoid cell line (LCL), growing in clumps and with irregular cell shapes. SPIBL-II and III showed many features of Burkitt-lymphoma derived cell lines, with rounded cells not forming clumps. Moreover the karyotype of SPIBL-I, the LCL-like cell was close to diploid, while SPIBL-II and III were close to tetraploid with a lot of aneuploidy, with several marker chromosomes: chromosome number 1, 3, 6, 7, 11, and 15.

**Table 2:** Characterization of cell lines

**Cell line**	**Date/biopsy (yymmdd)**	**Source**	**HIV-1**	**HHV-8**	**EBV**	Ig-rearrangement*	**Ig-expression**	**Phenotype**	**Karyotype/aberration in chromosome no**
I	971001	Ascites	-	-	+/ latency III	+ 87-92kD	+ kappa	LCL	Diploid
II	980526	Pleura	-	-	+/ latency I	+ 87–92 kD/108 kD	+ kappa	BL	Chr 1, 3, 6, 7, 11 and 15.
III	980626	Pleura	-	-	+/ latency I	+ 87-92kD/108 kD	+ kappa	BL	Chr 1, 3, 6, 7, 11 and 15.

* clonal rearranged Ig-heavy chain locus fragments detected by PCR.

The EBV gene expression patterns were consistent with these observations, SPIBL-I showed a latency III pattern and SPIBL-II and III both showed latency I pattern, expressing only EBNA 1, but no EBNA 2.

Clonal analysis was performed using the rearrangement of immunoglobulin heavy chain genes. This analysis showed that all three cell lines are monoclonal, but that SPIBL-I have a different clonal origin (one restriction fragments at 87–92 kD) than SPIBL-II and III (one fragment at 87–92 kD and one at 108 kD), which both have the same clonal origin.

## Discussion

The clinical features of this patient’s lymphoma manifestations were at the time of diagnosis consistent with primary effusion lymphoma (PEL), with ascitic and pleural effusions. Gene expression profile analysis of PEL has suggested a plasmablastic derivation in common with PBL [15]. Nevertheless the biopsies and the derived cell lines were HHV-8 negative while they expressed EBV genes. In the recent WHO classification of malignant lymphomas [2] the plasmablastic type of lymphoma (PBL) was distinguished from other types of aggressive B-cell derived lymphomas. This classification, which is based on the combined clinical, morphological, and genetic and phenotypic features considers the PBL as an entity distinct from PEL and other immunodeficiency associated lymphomas. The key distinguishing characteristics of PBL are positivity for EBV, plasmacell-markers CD38 and CD138 and clonal cytoplasmic immunoglobulin but negativity for CD45 and HHV-8. In spite of a PEL-like presentation our current case fulfills the criteria above for a PBL.

HIV-associated PBL is considered as a very aggressive lymphoma, with a poor prognosis also in the era of cART [16].

As far as could be analyzed on the autopsy material this lymphoma had histopathological and phenotypical features that were similar at first diagnosis and after recurrence. At relapse the lymphoma was spread both to extra-nodal sites and reappearing in effusions.

The CD4 expression on the PBL cells was unexpected, but has been seen in other cases, as has recently been reviewed [17]. Moreover, in a recent publication Goto H et al. [18] describes the establishment of a CD4-positive cell line from an HIV-patient with PEL making our finding less conspicuously unique.

The cell lines derived from the biopsies taken before and after recurrence were distinctly different. Cell lines from the early lymphoma had a lymphoblastoid cell line feature, i.e. similar to immunoblastic lymphoma. The cell lines derived from pleural effusion at recurrence were BL like, but did not have the t(8;14) translocation typical for BL. They expressed EBV-latency program I/II, which altogether would be compatible with the plasmablastic phenotype, also suggested by other markers on the cell lines. The cell lines were also different from reported HHV-8-positive PEL lines, which regularly do not express any immunoglobulin [3].

From clonal analysis of immunoglobulin-rearrangement we can conclude that the cell lines seem to share one marker of rearrangement of the heavy chain locus, while another marker is seen in addition to the BL like/PBL-cell lines. This might indicate that they represent clonal progression from the original lymphoma precursor cell, although it is more likely that they are derived from different clones. SPIBL-I could also be a spontaneous lymphoblastoid cell line derived from latently EBV infected cells not related to the tumor, although its origin from the ascitic effusion would be compatible with a tumor origin.

At the time of progression the HIV-RNA was non-detectable and CD4+ cell count had increased, although not to an acceptable level. A persevering decreased CD4+ cell count can be reflected in the lack of EBV control [19]. A long lasting CD4 cell decline together with increased EBV load seems to be an indicator of lymphoma risk. In spite of the introduction of cART to patients with such durable CD4+ cell depletion, the normalization of CD4+ cell number and function takes a considerable period of time [20]. EBV-DNA load is not a direct indicator of lymphomagenesis [21] in HIV-1 infected patients although they may show elevated EBV-DNA values prior to the lymphoma diagnosis [22].

Our finding of an EBV-DNA decline and persistence of low EBV-DNA loads clearly reflects the cytostatic treatment effect on the lymphoma. The subsequent, pronounced EBV-DNA increase preceding the lymphoma relapse suggests a value of EBV monitoring in treatment as well as follow up of EBV-positive lymphomas. This indicates the use of EBV-DNA load as a marker for recurrence as well as a treatment effect marker. The use of EBV-DNA load has also been clearly shown in the assessment of risk for EBV-positive PTLD in transplanted patients [23-25].

In HIV-1-infected patients a long lasting decline of CD4+ cells will disrupt the host-EBV balance and increase the risk for EBV related malignancy. In spite of the worldwide access to cART and awareness of HIV-1 there are still a considerable number of undiagnosed HIV-1-infected individuals, who will have developed severe immune deficiency at the time of HIV diagnosis. When initiating cART treatment at the time of HIV-diagnosis in this patient group (so called “late testers”), repeated analysis of EBV- could be of value in estimating the lymphoma risk.

## Conclusion

In this rare PBL with effusion our finding of EBV-DNA decline and persistence of low EBV-DNA load reflects the positive effect of cytostatic treatment on the lymphoma. This finding together with a pronounced EBV-DNA increase preceding the lymphoma relapse demonstrates the value of EBV monitoring in treatment of EBV-positive lymphomas in AIDS-patients. The result indicates the usefulness of EBV-DNA load as a marker for recurrence and treatment effects. EBV DNA-load might also be useful as an indicator of lymphoma risk in HIV-infected patients presenting late in HIV-1 infection with severe immunodeficiency.

## Methods

### Patient

A 36 year old homosexual man was diagnosed with HIV-1 in 1993. Two years later a Kaposi conjunctival lesion was removed (Figure 1). The second year, with a CD4+ cell count of 30 × 10^6^/L, antiretroviral treatment was initiated and in year three the patient began with cART. In the middle of year four the patient was diagnosed with a lymphoma. The lymphoma went into remission after cytostatic treatment, but recurred three months later with massive pleural effusion. The patient died in middle of year five. Biopsies were taken from the gut the first year, four years prior to the lymphoma diagnosis and from the developed lymphoma on three occasions in year five.

### Morphologic, immunohistochemical and flow cytometric analysis

The biopsies and autopsy specimens were fixed in neutral buffered formalin and processed for routine histopathological analysis by histochemical staining of paraffin sections.

Immunohisto- and cytochemistry was done according to standardized procedures and with reagent kits using manual and automated processes (Techmate 500 plus immunostainer, Dakopatts, Glostrup, Denmark). For immunocytochemistry an alkaline phosphatase chromogenic substrate was used, whereas for immunohistochemistry a peroxidase-based detection system was employed according to the manual (Dako ChemMate Detection kits; Peroxidase/diaminobenzine and a Rabbit/Mouse polylinker). Pre-treatment protocols for antigen demasking on paraffin sections were used according to standard procedure for each antibody.

Flow cytometry was performed with a Becton-Dickinson analyser (Becton-Dickinson).

### Establishment and culture of cell lines

Tissue biopsy materials, aspiration from ascitic and pleural effusions, were collected upon surgical excision or aspiration from the patient, stored in sterile 0.9% NaCl and were set up within 4 hours for tissue culture. Biopsy material was minced with scissors and forceps and passed through a metal wire mesh 3–4 times. The cell suspension thus generated was set up in 12-well microplates with RPMI 1640 and antibiotics. The cultures were followed regularly by microscopical examination and fed by replacing some half the culture medium once every 4–7 days in the beginning. Cells from the first biopsy occasion were transferred to microplates with irradiated fibroblast feeder layer. After one-two weeks clumps or loose aggregates of cells were detected and they were explanted in five mL Falcon plastic tubes on feeder layers after 2–3 months, and subsequently upon expansion of the cells to 25 mL Falcon bottles (spontaneous immunoblastoid cell line, SPIBL I). The second and third attempts to establish cell lines (SPIBL II and III) from the relapsed pleura effusions were very different and cells grew out much faster than on the first attempt, already 1–2 weeks after in vitro explanation. The established cell lines were used for characterization and also stored away by freezing.

### Phenotypic analysis of cell lines

The cell lines were analyzed by standard light microscopy.

### Analysis of karyotypes

Chromosomes in the cell lines were analyzed by making metaphase plates and stained for Giemsa-banding, according to Zech et al. [26].

### Analysis of immunoglobulin-gene rearrangement

Cellular DNA was analyzed using a PCR method [27].

### DNA and RNA quantification

EBV-PCR from blood analysis and statistics were performed as previously reported by Friis et al. [19]. EBV was measured in purified B-cells to get comparable values between time points, avoiding the confounder of variation in B-cell proportion of as well as the effect of B-cell variation over time in AIDS patients [19]. Quantification of HIV-1 RNA levels in blood were determined by using commercial kits NASBA (BioMérieux) or Amplicor HIV-1 monitor test (version 2.0, Roche Diagnostic Systems Inc.) and performed as recommended by manufacturer.

## Abbreviations

EBV: Epstein-Barr virus; cART: Combined anti-retroviral treatment; HIV-1: Human immunodeficiency virus 1; HHV-8: Human herpes virus number 8; PEL: Primary effusion lymphoma; PBL: Plasma blastic lymphoma; SPIBL: Spontaneous immunoblastoid cell line.

## Competing interests

No commercial relationship or potential conflict of interest related to the submission exists. No non-financial competing interests exist.

## Authors’ contributions

AF participated in the design of the study, carried out EBV laboratory work and wrote manuscript. BÅ participated in the design of the study, recruited patient and wrote manuscript. KG participated in the design and coordination of the study, recruited patient and wrote manuscript. AA carried out sample preparation and cell line related laboratory work. IE participated in the design of the study, wrote manuscript. BC made pathology evaluations and wrote manuscript. JZZ carried out HHV-8 laboratory work. All authors read and approved the final manuscript.
